# Structure and Magnetic Properties of Iron Thin Films Prepared at Different Deposition Times

**DOI:** 10.3390/ma19010165

**Published:** 2026-01-02

**Authors:** Chunxia Zhou, Liang Yan, Biao Yan, Zhiya Han

**Affiliations:** 1 School of Materials, Shanghai Dianji University, Shanghai 201306, China; zhiyahan@sdju.edu.cn; 2 School of Intelligent Manufacturing and Control Engineering, Shanghai Polytechnic University, Shanghai 201209, China; 3 School of Material Science and Engineering, Tongji University, Shanghai 201804, China

**Keywords:** iron thin film, deposition time, crystal structure, surface morphology, soft magnetic properties

## Abstract

In this paper, a series of iron thin films were prepared using the direct current magnetron sputtering method at different deposition times. By means of characterization techniques such as X-ray diffraction (XRD), transmission electron microscopy (TEM), atomic force microscopy (AFM), and vibrating sample magnetometer (VSM), the structure, surface morphology, and magnetic properties of the iron thin films prepared at different deposition times were systematically investigated. The XRD results indicate that all the iron thin films exhibit a polycrystalline body-centered cubic structure, with an obvious preferred orientation in the (110) direction. As the deposition time increases, the average grain size of the iron thin films gradually increases. This is mainly because the post-sputtered atoms can provide the energy required for the formation, movement, and growth of the already deposited grains or clusters. When the deposition time is too long, factors such as elastic effects and size constraints will limit the growth of grains and clusters. Therefore, for the thin films deposited after 120 s, the average grain size gradually stabilizes. When the deposition time is short, the thin films usually grow in the form of island-like accumulation. Grains and clusters of uneven sizes accumulate on the substrate, so the roughness gradually increases. This also implies an increase in the density of defects such as internal stress and vacancies within the thin film. As the deposition time increases, the thin films gradually transform to grow in a layered and flat manner, and the grain size gradually stabilizes and becomes relatively uniform. Therefore, the roughness of the thin film samples decreases and tends to be stable. The magnetic property test results show that all the iron thin films exhibit ferromagnetism. The iron thin film prepared at a deposition time of 120 s has the best comprehensive performance, with a saturation magnetization M_s_ of 1567 emu/cm^3^, a coercivity of 92 Oe, and a remanence ratio of 0.86.

## 1. Introduction

Iron thin films, fundamental to ferromagnetic thin-film materials, are extensively utilized in electrical and electronic devices. Their applications include magnetic recording, magnetoresistive sensing, electromagnetic switches, magnetic shielding, and spin semiconductors. This widespread use is attributed to their exceptional properties, such as high saturation magnetic induction and low coercivity [1].

Common methods for preparing ferromagnetic thin films include molecular beam vapor deposition [2], magnetron sputtering [3], cold spray technology [4], and ultrasonic-assisted electrodeposition [5]. Among these, magnetron sputtering is widely used due to its high film-forming speed, uniform and controllable film thickness, and excellent process repeatability. Researchers have extensively studied ferromagnetic thin films prepared using this technique. By incorporating elements like N and Co, the saturation magnetization, resistivity, and high-frequency magnetic permeability of the film can be enhanced [6]. Iron ion irradiation increases the lattice constant and grain size, resulting in higher magnetic moments at the top and bottom of the film compared to the bottom layer, effectively modifying the structure and magnetic properties of the film [7]. Annealing can alter the texture of the film [8] and reduce coercivity. Heat treatment in a hydrogen atmosphere can create an oxygen-free and silicon-free interface, further modifying the structure and magnetic properties of the film [9]. Serizawa et al. [10] used various substrates to grow iron films with different crystal orientations to adjust magnetostrictive behavior. While these methods can optimize or adjust the magnetic properties of the film, they may also introduce challenges. For instance, ion irradiation can damage the film surface, complicating the process and preparation, while doping with non-magnetic elements might reduce the film magnetization.

Optimizing growth parameters is a fundamental and effective method for enhancing thin-film properties. During thin-film preparation, sputtering parameters significantly impact the morphology, structure, and magnetic characteristics of the films. For instance, sputtering power influences the magnetic anisotropy of BiFeO_3_-CoFe_2_O_4_ nanocomposite thin films [11] and affects the surface morphology, grain size, and growth of Co_2_FeSi thin films, thereby altering their soft magnetic properties [12]. In TbFeCo thin films, coercivity can be adjusted by sputtering power [13], and increasing this power can change Tb-Fe thin films from superparamagnetic to ferromagnetic [14]. Under zero-field or field cooling, sputtering power also affects the composition and magnetization behavior of Co_2_FeSi thin films [15]. Numerous studies highlight the significant impact of working gas pressure on growth mode, structure, and properties. Ramírez et al. [16] demonstrated that altering working gas pressure allows Fe_1-x_Ga_x_ thin films to achieve a high magnetoelastic coefficient. Li et al. [17] showed that the magnetic anisotropy of FeCoB thin films can be rotated by 90° with changes in working pressure. Srinivas et al. [15] found that low working gas pressure enhances the surface morphology and magnetic properties of Co_2_FeSi thin films. Tiwari et al. [18] noted that reducing working gas pressure significantly increases the magnetoresistance of Fe-Cu-Ni. Mishra et al. [19] observed that increasing the thickness of Fe-Co-Al thin films enhances magnetostatic interactions between magnetic particles. Yang et al. [20] found that as thin film thickness increases, coercivity decreases significantly, and higher substrate temperatures improve the saturation magnetization of thicker films. Kumar et al. [21] studied the effect of ferromagnetic layer thickness on CoFeB thin films, finding that increased thickness enhances saturation magnetization and remanence ratio while reducing coercivity. Cao et al. [22] noted that as thin film thickness increases, film roughness rises, and both coercivity and remanence decrease. Despite these findings, few studies have explored the influence of sputtering parameters on the structure and magnetic properties of iron thin films prepared by magnetron sputtering. Thus, investigating how different sputtering parameters affect the growth mode, crystal structure, and magnetic properties of iron thin films holds significant theoretical and practical value. In our previous work, we examined the effects of sputtering power and working gas pressure on the structure and magnetic properties of iron thin films prepared by direct current magnetron sputtering.

This paper presents the preparation of a series of pure iron thin films using direct current magnetron sputtering. The study investigates how deposition time influences the crystal structure, surface morphology, and magnetic properties of these films. The goal is to determine the optimal preparation method and process parameters, offering theoretical guidance for regulating thin film properties.

## 2. Materials and Methods

**Figure 1:**
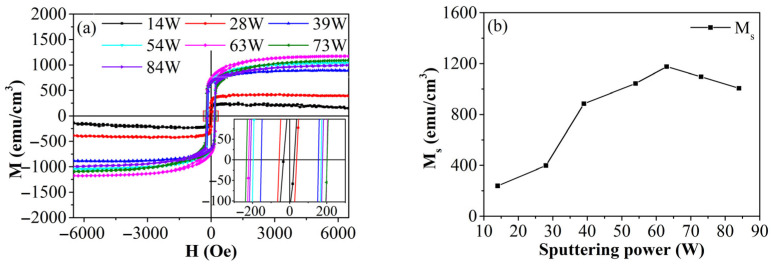
Magnetic properties of Fe films prepared at different deposition time: (**a**) hysteresis loop, (**b**) M_s_.

Iron thin film samples were deposited on single-crystal silicon substrates using direct-current magnetron sputtering. The target material, a high-purity patch (Ø60 mm, 99.99%), was supplied by Zhongnuo New Materials (Beijing, China) Technology Co., Ltd. (Beijing, China), while the single-crystal silicon (100) substrate came from Zhejiang Lijing Silicon Materials Co., Ltd. (Quzhou, China). The silicon was ultrasonically cleaned in absolute ethanol and acetone for 5 min each to remove surface grease before being placed on the rotating disk in the vacuum chamber. To ensure film purity and stable glow discharge, the chamber was evacuated to 10^−5^ Pa. The argon flow rate and working pressure were then adjusted for stable glow discharge of the target material, initiating the thin-film deposition. Based on previous research, the sputtering power was set at 63 W, as shown in Figure 1. The working pressure was set at 0.6 Pa, with deposition times ranging from 30 to 300 s, and the substrate remained unheated.

The composition and crystal structure of the thin-film samples were characterized using X-ray diffraction (XRD) (DX-2007, Dandong Fangyuan Co., Ltd., Dandong, China) and transmission electron microscopy (TEM) (JEM-2100F, JEOL Co., Ltd., Tokyo, Japan). Their magnetic properties were assessed with a vibrating sample magnetometer (VSM) (Model 7407, Lakeshore, Carson, CA, USA). Atomic force microscopy (AFM) (Dimension Icon, Bruker, Billerica, MA, USA) was employed to observe and analyze the microscopic morphology, surface roughness, and grain size of the samples. The thickness of the thin-film samples was tested and analyzed by X-ray reflectivity (XRR) (D8 ADVANCE, Bruker, Billerica, MA, USA). All tests were performed at room temperature.

## 3. Results and Discussion

### 3.1. Crystal Structure

**Figure 2:**
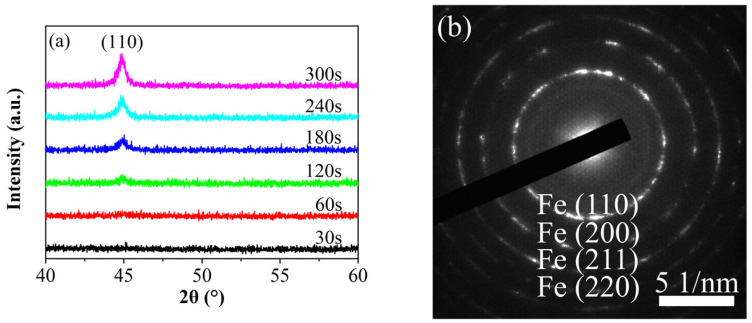
XRD patterns (**a**) of Fe film prepared at different deposition time and TEM (**b**) of Fe film at 120 s.

**Figure 3:**
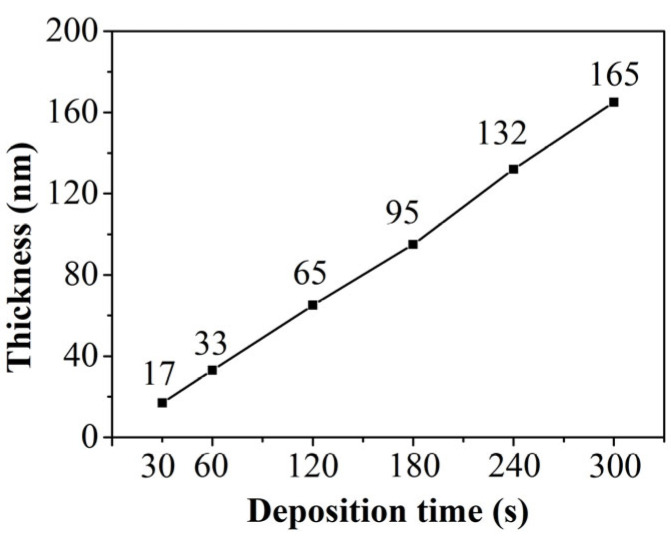
Line charts of the thickness of Fe films as a function of the deposition time.

Figure 2a presents the XRD patterns of iron thin films prepared at various deposition times. The results reveal that thinner films lack distinct diffraction peaks, primarily due to their high signal-to-noise ratio and the equipment limited measurement accuracy. As the deposition time increases, the thickness of the thin film shows a linear growth, as shown in Figure 3. Diffraction peaks in the (110) direction gradually appear in the iron thin film, and their intensities gradually increase. Given the small thickness of the film, all samples exhibit a pronounced preferred orientation in the (110) direction. To further confirm the crystal structure, TEM observations were conducted on selected samples, as depicted in Figure 2b. The figure clearly shows that the films possess evident polycrystalline characteristics.

### 3.2. Surface Morphology

**Figure 4:**
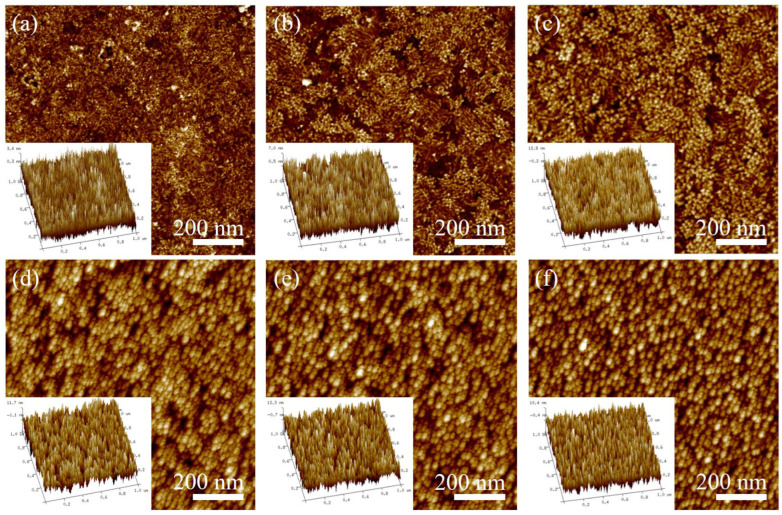
AFM micrographs of Fe films at different deposition time: (**a**) 30 s, (**b**) 60 s, (**c**) 120 s, (**d**) 180 s, (**e**) 240 s, (**f**) 300 s.

Figure 4a–f illustrate the surface morphology maps and their three-dimensional views of iron thin-film samples prepared at varying deposition times. The morphology maps reveal significant changes in the surface morphology of the iron thin film as deposition time increases. At a deposition time of 30 s, fine grains stack and grow on the substrate, with gullies, or darker-colored holes, visible despite the absence of an obvious island-like structure. This suggests that with shorter deposition times, the thin film grows in an island-like manner, gradually covering the substrate. At 60 s, the grains grow similarly, but both grain size and clusters become larger. This occurs because the sputtered iron atoms possess heat, providing energy for the growth of already-deposited iron atoms, thereby promoting grain growth. The figure also shows numerous defects, such as holes, on the thin-film surface. As deposition time increases, the sputtered atoms continuously supply kinetic energy for the formation, movement, and growth of previously deposited atoms. Consequently, the iron thin film prepared at 120 s exhibits larger grain size and a denser structure. However, as deposition time further increases (180 s, 240 s, 300 s), grain size does not increase indefinitely. This is primarily due to elastic effects and size constraints, causing grain size to stabilize under high deposition amounts. If deposition time continues to increase while maintaining this growth mode and particle size, the thin film grows stably, but defects like internal stress and holes accumulate exponentially.

**Figure 5:**
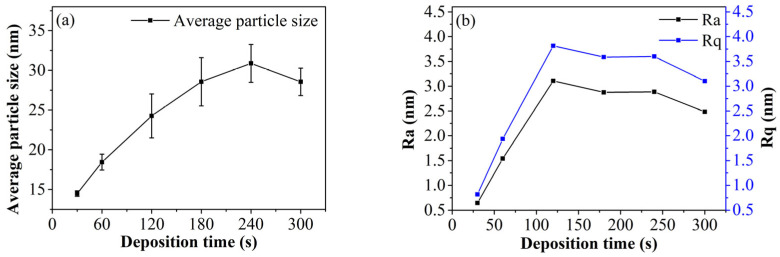
The particle size (**a**) and the surface roughness (**b**) of iron films at different deposition time. (Ra: Arithmetic Average Roughness; Rq: Root Mean Square Roughness).

Figure 5 illustrates the average particle size and roughness of the iron thin film. As shown in Figure 5a, the average particle size increases with deposition time and eventually stabilizes. This trend aligns with the changes observed in surface morphology. As previously mentioned, increased deposition time allows incident particles to supply energy for the formation, movement, and growth of the atomic layer. Thus, within a certain time frame, the average particle size exhibits a linear growth trend with deposition time. However, factors such as elastic effects and size constraints cause the average particle size to stabilize. For instance, the average particle sizes for samples deposited at 180 s, 240 s, and 300 s are quite similar. This stabilization also explains why, in the later stages of deposition, the roughness fluctuations decrease and stabilize, as depicted in Figure 5b. Initially, the thin film grows primarily in an island-stacking mode. As deposition time increases, the average particle size and surface roughness both increase. In the later stages, the film grows in a layered mode, leading to stabilization in both particle size and roughness.

### 3.3. Magnetic Properties

**Figure 6:**
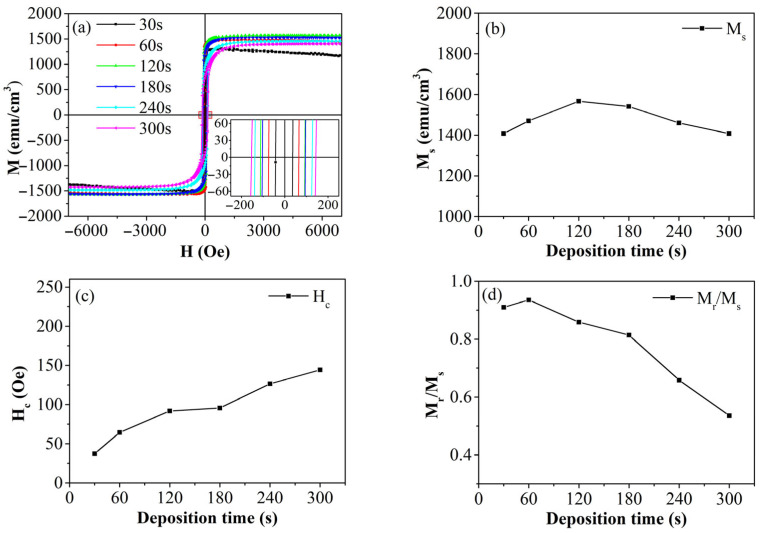
Magnetic hysteresis loops of Fe films at different deposition time (**a**); saturation magnetization (M_s_) (**b**), coercivity (H_c_) (**c**) and squareness ratio M_r_/M_s_ (**d**) of Fe films prepared at different deposition time.

In this study, we measured the in-plane magnetic hysteresis loops of iron thin films prepared under varying deposition times, as illustrated in Figure 6a. The figure clearly demonstrates that all thin-film samples exhibit strong ferromagnetic properties. From the magnetic hysteresis loops, we derived the trends in saturation magnetization (M_s_), coercivity (H_c_), and remanence ratio (M_r_/M_s_) as functions of deposition time, as depicted in Figure 6b–d. The magnetic property parameters of the iron thin films in this study and previous studies are presented in Table 1.

Figure 6b shows that the M_s_ values of all iron thin film samples exceed 1300 emu/cm^3^, highlighting their excellent soft magnetic properties. Although the M_s_ values do not vary significantly with increased deposition time, there is a noticeable trend: they initially rise and then fall. Notably, the iron thin film deposited for 120 s achieves the highest M_s_ value, reaching 92% of bulk iron. Initially, the thin film grows in an island-like mode with low thickness and density. As deposition time increases, iron atoms gradually cover the substrate, transitioning the growth to a more compact layer-by-layer mode. Concurrently, the grain size increases as the layers accumulate. However, at higher deposition amounts, grain size stabilizes due to elastic effects and size constraints, as observed in the AFM analysis. The film deposited for 210 s exhibits optimal M_s_. Yet, prolonged deposition times and high energy accumulation introduce defects and inhomogeneities, increasing grain size variability and defect density, which degrade performance. Thus, deposition time significantly influences the saturation magnetization of thin films.

Figure 6c illustrates the coercivity of iron thin films at varying deposition times. The data reveal that H_c_ progressively rises with longer deposition times, indicating a corresponding increase in internal stress and defect density within the film. As deposition time extends, the film thickens, resulting in a greater number of stacked iron atomic layers. This stacking, along with the layer-by-layer growth of grain size, contributes to accumulating internal stress and a higher density of defects such as vacancies and dislocations. These defects create a pinning effect during the magnetization process, impeding the rotation of magnetic domains and consequently raising coercivity. Thus, deposition time significantly influences the coercivity of thin films.

As deposition time increases, the M_r_/M_s_ of thin film samples gradually decreases, as illustrated in Figure 6d. With longer deposition times, the thickness of the thin film also increases. This increase in thickness leads to a rise in both the saturation field and the switching field of the iron thin film, while the remanence ratio decreases. The remanence ratio is influenced by factors such as the demagnetizing field and stress. As thickness grows, the demagnetizing field ratio of the thin film samples decreases, while internal stress and defect density rise, contributing to a reduction in the remanence ratio. Consequently, the remanence ratio of the thin film exhibits a downward trend.

## 4. Conclusions

In this paper, we successfully prepared a series of iron thin films using the direct current magnetron sputtering method. We investigated the crystal structures, surface morphologies, and magnetic properties of these thin-film samples for various deposition times. XRD results reveal that all iron thin films possess a polycrystalline body-centered cubic structure with a pronounced preferred orientation in the (110) direction, which directly contributes to their ferromagnetism. As deposition time increases, the average grain size of the iron thin films also increases. This occurs because post-sputtered atoms provide the necessary energy for the formation, movement, and growth of already deposited grains or clusters. However, when deposition time is excessive, factors such as elastic effects and size constraints restrict further growth of grains and clusters. Consequently, for films deposited beyond 120 s, the average grain size stabilizes. With shorter deposition times, thin films typically grow in an island-like accumulation, leading to unevenly sized grains and clusters on the substrate, which increases roughness. This roughness implies a higher density of defects, such as internal stress and vacancies, within the thin film. As deposition time extends, the films gradually transition to a layered and flat growth pattern, resulting in more uniform grain sizes and reduced roughness. Magnetic property analysis indicates that the iron thin film deposited at 120 s exhibits the best overall performance, featuring the highest saturation magnetization (M_s_), along with moderate coercivity and remanence ratio.

## Figures and Tables

**Table 1:** Magnetic properties of Fe thin films under different preparation conditions.

Samples	Methods	M_s_ (emu/cm^3^)	H_c_ (Oe)	M_r_/M_s_	References
30 s	DC magnetron sputtering	1407	37	0.91	This research
60 s		1470	65	0.94	
120 s		1567	92	0.86	
180 s		1541	96	0.81	
240 s		1460	127	0.66	
300 s		1408	144	0.54	
230 W	RF magnetron sputtering	1566	112	0.40	[3]
Rt 0 T	Thermal evaporation	1213	58	-	[23]
0 T	Molecular beam deposition	1491	-	0.54	[1]

## Data Availability

The original contributions presented in this study are included in the article. Further inquiries can be directed to the corresponding authors.
